# Ultrastructural and Cytological Studies on *Mycosphaerella pinodes* Infection of the Model Legume *Medicago truncatula*

**DOI:** 10.3389/fpls.2017.01132

**Published:** 2017-06-30

**Authors:** Tomoko Suzuki, Aya Maeda, Masaya Hirose, Yuki Ichinose, Tomonori Shiraishi, Kazuhiro Toyoda

**Affiliations:** ^1^Laboratory of Plant Pathology and Genetic Engineering, Graduate School of Environmental and Life Science, Okayama UniversityOkayama, Japan; ^2^Faculty of Science, Japan Women’s UniversityBunkyo-ku, Japan

**Keywords:** transmission electron microscopy (TEM), disease resistance, energy-dispersive X-ray (EDX) spectroscopy, hydrogen peroxide (H_2_O_2_), intrahyphal hyphae, susceptibility

## Abstract

Ascochyta (Mycosphaerella) blight on cultivated peas is primarily caused by infection through asexual spores (pycnospores) of *Mycosphaerella pinodes* (Berk. et Blox.) Vestergren [recently renamed *Peyronellaea pinodes* (Berk. & A. Bloxam) Aveskamp, Gruyter & Verkley]. Using a model pathosystem involving *Medicago truncatula* and *Mycosphaerella pinodes* strain OMP-1, we examined the histology and ultrastructure of early infection events and fungal development including penetration by appressoria, vegetative growth of infection hyphae, and host responses. On the susceptible ecotype R108-1, pycnospores germinated and grew over the surface of the epidermis, then formed an appressoria and penetrated the cuticle. Beneath the cuticle, the infection peg expanded into a hyphae that grew within the outer wall of the epidermis. Subsequently, the hyphae penetrated down within mesophyll cells and proliferated vigorously, eventually, forming asexual fruiting bodies (pycnidia). In contrast, successful penetration and subsequent growth of infection hyphae were considerably restricted in the ecotype Caliph. Detected by its reaction with cerium chloride (CeCl_3_) to generate electron-dense cerium perhydroxides in transmission electron micrographs, hydrogen peroxide (H_2_O_2_) accumulated in epidermal and mesophyll cells of Caliph challenged with pycnospores of *M. pinodes*. This intracellular localization was confirmed by energy-dispersive X-ray spectroscopy. Our observations thus indicate that the oxidative burst reaction leading to the generation of reactive oxygen species is associated with a local host defense response in Caliph, since no clear H_2_O_2_ accumulation was detectable in susceptible R108-1. Indeed, aberrant hyphae such as intrahyphal hyphae and dead hyphae, probably due to a local defense elicited by the fungus, were abundant in Caliph but not in R108-1. Our results on the cellular interactions between the fungus and host cells provide additional insights to understand foliar infection by *M. pinodes* on cultivated peas.

## Introduction

Ascochyta (Mycosphaerella) blight of pea, caused by *Mycosphaerella pinodes* (Berk. et Blox.) Vestergren [syn. *Peyronellaea pinodes* (Berk. & A. Bloxam) Aveskamp, Gruyter & Verkley], is one of the most important diseases of grain legumes worldwide, especially in Europe, North America, Australia, and New Zealand (Moussart et al., 1998; Bretag and Ramsey, 2000). The disease annually causes 10% yield losses and occasionally reach 50% yield losses (Wallen, 1965, 1974; Xue et al., 1997). Despite the economic impact and numerous studies on this disease, little is known about the cytological features during infection by *M. pinodes*, especially in resistant interactions. One reason is due to the lack of resistant cultivars of pea (*Pisum sativum* L.) as well as the available resources in the *Pisum* germplasm collection with strong resistance to this disease (Prioul-Gervais et al., 2007).

*Mycosphaerella pinodes* is a hemibiotrophic pathogenic fungus that directly penetrates host epidermal cells. This disease is normally initiated by asexual pycnospores, which germinate to develop non-melanized appressoria that penetrate host cuticles directly (Clulow et al., 1991; Nasir et al., 1992). Clulow et al. (1991) reported that a pycnospore of *M. pinodes* germinated to form a germ tube which differentiates into an appressorium that directly penetrates the host cuticle. Subsequently, the infection peg formed an infection hyphae, which grows through the outer wall of the epidermis without killing the epidermal cells, frequently penetrating cells directly. Based on microscopic observations, they suggested that the early stage of infection, lasting at least 48 h after inoculation, is biotrophic and is then followed by the typical necrotrophic phase involving progressive necrosis.

In regard to fungal virulence and disease development, we found that this fungus secretes both an elicitor and a suppressor for plant defense during germination, mainly before the actual penetration (Shiraishi et al., 1978, 1992). Shiraishi et al. (1992) successfully determined the chemical structures of two suppressors, named supprescins A and B. These are small mucin-type glycopeptides containing *N*-acetylgalactosamine (NAcGal) attached to the serine residue in the peptide moiety. Interestingly, the supprescin B exhibits a “V-shaped structure” with a strong positive charge, which readily facilitates targeting of the host protein(s) (Shiraishi et al., 1992, 1997; Toyoda et al., 2016). In fact, the purified supprescins can severely inhibit the proton-pumping activity of host plasma membrane ATPase (Yoshioka et al., 1990; Shiraishi et al., 1991; Amano et al., 1995) and the related signal transduction pathway dependent on phosphatidylinositols and related lipids (Toyoda et al., 1992), temporarily reducing the capability of the host cell to defend itself (Yamada et al., 1989). Actually, the suppressor treatment renders the host cells susceptible even to unrelated (non-pathogenic) pathogens (Shiraishi et al., 1997; Toyoda et al., 2011, 2016), indicating that the suppressors are required for conditioning susceptibility of host cells.

The oxidative burst is one of the earliest defense responses to pathogen attack which leads to a transient accumulation of reactive oxygen species (ROS), including superoxide (O_2_^-^), hydrogen peroxide (H_2_O_2_), and hydroxyl radical (Bradley et al., 1992; Mehdy, 1994; Lamb and Dixon, 1997). ROS produced during the oxidative burst not only protect against invading pathogens, but also act as signaling molecules that initiate plant defense responses (Bradley et al., 1992; Mehdy, 1994; Lamb and Dixon, 1997). In some cases, ROS can inhibit pathogen growth by strengthening host cell walls through oxidative cross-linking of glycoproteins, such as proline-rich protein (Bradley et al., 1992; Deepak et al., 2007). Previously, we demonstrated that the oxidative burst in pea leaves elicited with an elicitor preparation from *M. pinodes* is effectively inhibited or delayed by a suppressor from the same fungus (Kiba et al., 1996, 1997; Toyoda et al., 2012; Amano et al., 2013). On the basis of these findings, it is likely that a rapid and effective production of ROS is a hallmark of resistance response to the fungal infection. However, subcellular aspects of host defense responses to *M. pinodes* infection, especially in relation to resistance have been studied little. The purpose of this study was thus to observe infection behavior after germination of pycnospores of *M. pinodes* as well as the host cell responses to the infection, using a recently developed model pathosystem involving *Medicago truncatula* (Toyoda et al., 2013a). Specifically, we aimed to observe the ultrastructural features during symptom development and differences in host responses between *M. truncatula* ecotypes. Fungal strategies resulting in successful colonization are also discussed.

## Materials and Methods

### Plant Material and Growth Conditions

Previously, we evaluated disease susceptibility of 19 *Medicago truncatula* ecotypes to *Mycosphaerella pinodes* strain OMP-1 and selected ecotype R108-1 as a highly susceptible ecotype and Caliph as having the lowest susceptibility (Toyoda et al., 2013a). Seeds of *M. truncatula* ecotype R108-1 and Caliph were scarified by treatment with anhydrous sulfuric acid for 5 min, then washed thoroughly with tap water (Toyoda et al., 2013a). Seeds were then germinated on wetted filter paper, then grown on water-swelled Jiffy-7 peat pellets (AS Jiffy Products, Oslo, Norway) in a growth room at 22°C, with a 10 h light/14 h dark cycle at 11.8 W⋅m^-2^ as described previously (Toyoda et al., 2013a). Detached leaves of 4- to 6-week-old seedlings were used for all experiments.

### Pathogens and Inoculations

*Mycosphaerella pinodes* strain OMP-1 (NBRC 30342, ATCC 42741), isolated in Akaiwa City, Okayama Prefecture, Japan in 1978, was cultured onto V8 juice agar medium at 23°C for 7 days as described previously (Shiraishi et al., 1978). Pycnospores that formed were suspended in sterile distilled water, and the concentration was adjusted to 1 × 10^4^, 10^5^ or 10^6^ spores/ml for each inoculation. To perform a detached leaf assay, trifoliate leaves were excised from 4- to 6-week-old seedlings and maintained alive on moist cotton in a plastic tray filled with wetted paper towel. For inoculation, 10 μl of pycnospore suspensions containing 0.02% (v/v) Tween 20 was carefully dropped on the adaxial surface of detached leaves. Each tray was then covered with a clear plastic wrap to maintain a high humidity and kept in a dew chamber at 22°C, with 14-h illumination per day at 11.8 W⋅m^-2^.

### Light Microscopy

Pycnospore germination and subsequent formation of infection hyphae were observed with a light microscope (Olympus BX60, Olympus, Tokyo, Japan). The inoculated leaves were fixed with a mixture of ethanol and acetic acid (24:1, v/v) at 3, 6, 9, 12, and 24 hour post inoculation (hpi), and decolorized with the same mixture at room temperature for 6 h and stained with 0.5% (w/v) aniline blue (Nacalai Tesque, Tokyo, Japan) in 0.1 M potassium phosphate buffer (pH 8.5) for 15 min. The samples were then rinsed with 70% ethanol and distilled water and viewed with a bright field microscope (Olympus BX60, Olympus).

### DAB Staining

The inoculated leaves were soaked in 1 mg/ml 3,3′-diaminobenzidine (DAB) (Sigma–Aldrich, St. Louis, MO, United States) in distilled water at 18 or 24 hpi, and then vacuum-infiltrated for 30 s three times and incubated for an additional 8 h at room temperature. The leaves were then fixed and decolorized with the 24:1 mixture of ethanol and acetic acid at room temperature for 6 h. In most cases, the fungal structures were stained with aniline blue as described already, then viewed with the light microscope.

### Light and Transmission Electron Microscopy (TEM)

At 1, 3, 5 and 7 days post inoculation (dpi), the inoculated leaves were cut into small pieces (2 mm × 3 mm) with a razor blade. Samples of R108-1 and Caliph at 1 and 3 dpi were cut from the region beneath the inoculated site. Samples of R108-1 were prepared at 5 and 7 dpi from the region surrounding the inoculated site. The specimens were prefixed with 2.5% (v/v) glutaraldehyde in 0.1 M sodium phosphate buffer (pH 7.4) at 4°C overnight and postfixed with 1% (w/v) buffered osmium tetroxide at room temperature for 1 h. The fixed specimens were dehydrated in a graded ethanol series (30, 50, 70, and 90% v/v ethanol; 20 min each change and then three 30-min changes of 100% ethanol) and infiltrated with Quetol 651 resin mixture (Nissin EM, Tokyo, Japan). Semithin sections (700 nm) were cut from resin blocks using a diamond knife. The sections were mounted on a glass slide and stained with 0.6% (w/v) toluidine blue including 1% (w/v) sodium tetraborate. After washing with distilled water, the stained sections were observed with the light microscope (Olympus BX60). Ultrathin sections (70–90 nm) were cut from the resin blocks and mounted on copper grids. The sections were stained with 1% (w/v) uranyl acetate for 30 min and lead solutions for 30 min as described previously (Suzuki et al., 2003). Sections were observed with a transmission electron microscope (TEM) (H-7500, Hitachi, Tokyo, Japan) at 80 kV. Fixatives, cacodylate buffer and osmium tetroxide used here are biohazard, so all procedures including weighing and solution preparation of chemicals were performed in a fume hood using protective clothing and gloves. Handling and waste disposal were carried out according to the Guidelines for the Management of Chemical Substances issued by the Japanese government.

### TEM Observation for H_2_O_2_ Accumulation

We used a histochemical analysis to detect hydrogen peroxide (H_2_O_2_) *in situ*, based on the generation of cerium perhydroxides as described by Bestwick et al. (1998). The inoculated portions of the leaves were cut into to small pieces (2 mm × 3 mm), then the leaf pieces were soaked in 50 mM MOPS/KOH (pH 7.2) containing 5 mM CeCl_3_ for 1 h after the vacuum infiltration. Leaf pieces were prefixed in 2.5% (v/v) glutaraldehyde in 100 mM sodium cacodylate buffer (CAB) at room temperature for 4 h, and postfixed with 1% (w/v) buffered osmium tetroxide for 1 h. After fixation, leaf pieces were washed three times for 10 min in CAB and dehydrated with ethanol and infiltrated in Quetol 651 resin mixture as described above. The resin blocks were sectioned (100–120 nm) using a diamond knife, and the unstained sections were observed with the Hitach H-7500 TEM at 60 kV. Uninoculated leaf pieces were used as the control. For the negative control, inoculated leaf pieces were treated with MOPS as a substitute for CeCl_3_.

### Elemental Analysis for H_2_O_2_-Reactive Deposits

Intracellular localization of cerium perhydroxides resulting from reaction of H_2_O_2_ with CeCl_3_ was assessed cytochemically by energy-dispersive X-ray (EDX) spectroscopy. Unstained sections were subjected to EDX point microanalysis and EDX elemental mapping using a TEM and STEM system (H-7510, Hitachi) equipped with an EDX detector operated at 80 kV and analytical software (EMAX 5770W, Horiba, Kyoto, Japan).

## Results

### Infection Behavior of *M. pinodes* on *M. truncatula* Ecotype R108-1 and Caliph

**FIGURE 1:**
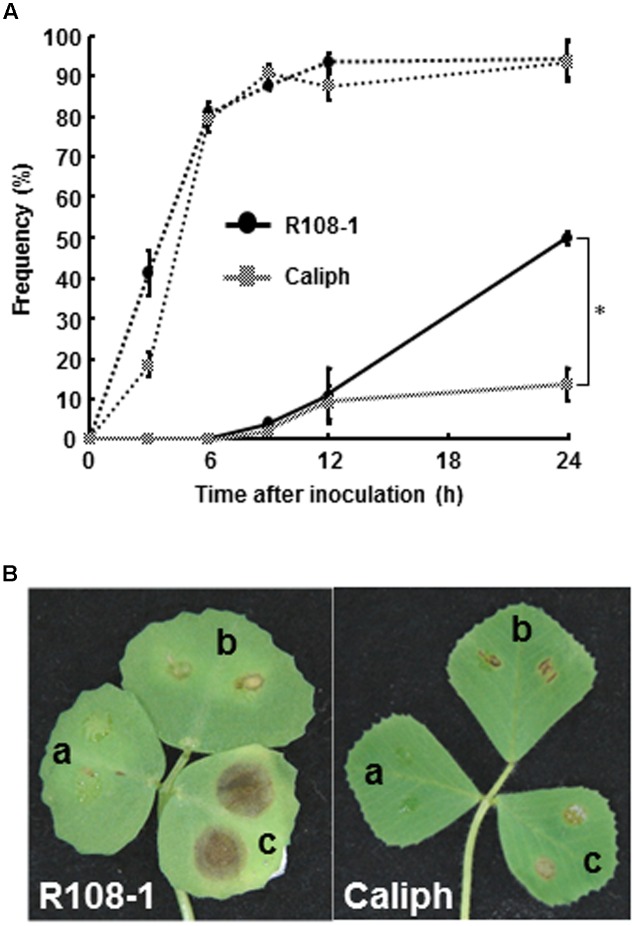
**(A)** Percentage germination of pycnospores (dotted line) and formation of infection hyphae (solid line) on/in detached leaves of *Medicago truncatula* ecotypes Caliph and R108-1. Values are means ± standard deviations from three independent experiments. Asterisk indicates a significant difference as revealed by a Student’s *t*-test. **(B)** Lesion formation on detached leaves of each ecotype after inoculation with pycnospores of *Mycosphaerella pinodes* (a = 1 × 10^4^, b = 1 × 10^5^, c = 1 × 10^6^ spores/ml) and incubation at 22°C for 3 days.

To observe infection and disease symptoms on leaves of two *M. truncatula* ecotypes (highly susceptible ecotype R108-1 and least susceptible ecotype Caliph), the leaves of both ecotypes were inoculated with pycnospores of *M. pinodes*. Repeated inoculation trials indicated that the percentage of pycnospore germination on both ecotypes was similar and that 80% of the pycnospores had germinated by 6 hpi (**Figure 1A**, dotted line). The rate of forming infection hyphae differed significantly between the two ecotypes. In particular, unlike on the R108-1, successful penetration was significantly suppressed on the Caliph, when assessed at 24 hpi (**Figure 1A**, solid line). Disease symptoms differed remarkably between the ecotypes especially when inoculated with a high concentration of pycnospores (1 × 10^6^ spores/ml). Brown lesions on R108-1 expanded from the inoculation sites, whereas those on Caliph never expanded (**Figure 1B**). Initial symptoms appeared as small, slightly raised spots on leaves, when R108-1 was inoculated with a low concentration of pycnospores (1 × 10^5^ or 1 × 10^4^ spores/ml) and the lesions were surrounded by yellow halos (**Figure 1B**, left). Roger et al. (1999) reported that spore germination, appressorial formation, and subsequent penetration were initiated on pea, by 2, 6 and 8 h after inoculation, respectively. Since spores germinated by 3 hpi and subsequent penetration through the wall of the epidermal cells occurred by 9 hpi on both R108-1 and Caliph of *M. truncatula* (**Figure 1A**), early infection events on the *M. truncatula* were quite similar to development on pea, except for the rate of forming infection hyphae in Caliph.

### Invasion Behavior of *M. pinodes* in Leaf Tissues of Both Ecotypes

**FIGURE 2:**
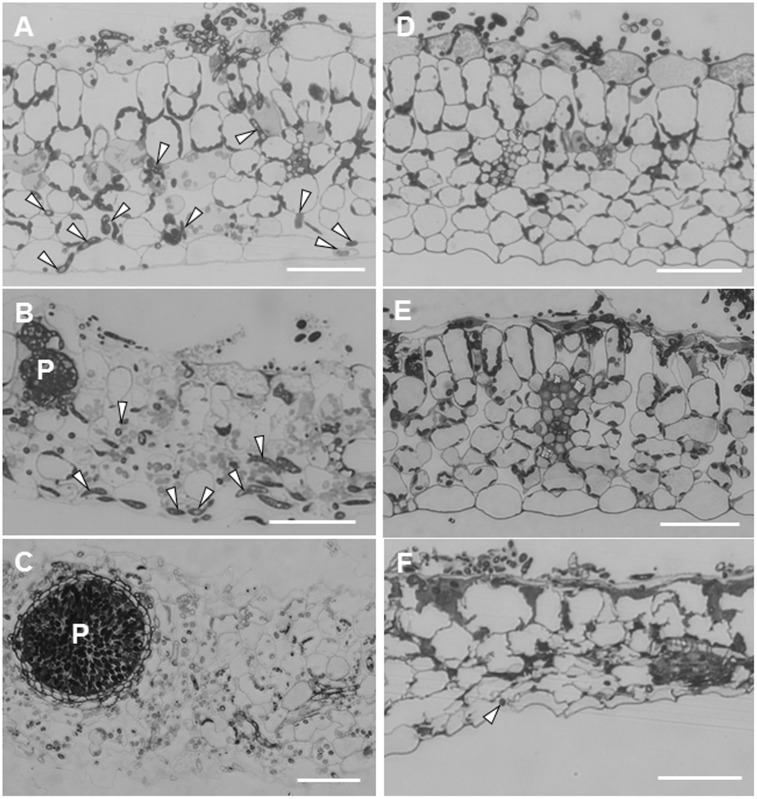
Transverse sections cut from leaves of *M. truncatula* ecotype R108-1 **(A–C)** and Caliph **(D–F)** inoculated with pycnospores of *M. pinodes*. All sections were stained with toluidine blue. **(A)** Infection hyphae (arrowheads) had penetrated the adaxial epidermis and grown to the abaxial epidermis by 1 day after inoculation (dpi). **(B)** At 3 dpi, infection hyphae proliferated within tissue underneath the inoculated region, and immature pycnidia (P) had formed. **(C)** At 5 dpi, infection hyphae had grown from the inoculated region, and a mature pycnidium (P) can be seen in the disintegrated leaf tissue. **(D)** At 1 dpi, infection hyphae had penetrated the adaxial epidermis but had not grown into mesophyll cells. **(E)** At 3 dpi, epidermal cells with infection hyphae collapsed, and infection hyphae had grown into certain mesophyll cells. **(F)** At 7 dpi, mesophyll cells as well as the abaxial epidermis was deformed, and cells were shrunken where sparse infection hyphae were observed at the abaxial epidermis. Bars = 50 μm.

Leaf sections showed striking differences in the extension of infection hyphae into leaf tissues between ecotypes R108-1 and Caliph. In susceptible R108-1, *M. pinodes* penetrated the adaxial epidermal surface (inoculated site) with appressoria and formed infection hyphae in the epidermal cells, and then grew intercellularly and eventually reached the abaxial epidermis (**Figure 2A**, arrowheads) just 1 dpi. At this stage, fungal structures were stained equally well as seen in healthy chloroplasts with toluidine blue, indicating that almost all plant cells retained their fine structure. At 3 dpi in R108-1 leaf tissues, infection hyphae remained entirely under the inoculated sites, and immature pycnidia often formed inside the mesophyll tissue (**Figure 2B**). The outline of the plant cells was amorphous, and the stained chloroplasts had disappeared as the result of plant cell disruption (**Figure 2B**). At 5 dpi, host cell organelles were completely disintegrated, and most pycnidia matured (**Figure 2C**). In R108-1, infection hyphae grew prolifically and reached the abaxial side of epidermis by 24 hpi, and subsequently formed pycnidia by 3 dpi (**Figures 2B,C**). The latent period for developing pycnidia on pea is 3–4 days (Roger et al., 1999), hence infection behavior of *M. pinodes* on the ecotype R108-1 is almost the same as seen on its natural host, pea.

Unlike on R108-1, the growth of infection hyphae after penetration in Caliph was restricted. At 1 dpi, infection hyphae had invaded only the adaxial epidermis, and cytoplasm in cells with hyphae were granulated (**Figure 2D**). At 3 dpi, although infection hyphae occasionally extended into palisade parenchyma cells, most host cells that contained hyphae had shrunk (**Figure 2E**, especially note the epidermal cells). Infection hyphae finally reached the abaxial epidermis (**Figure 2F**, arrowhead) at 7 dpi, and host tissues were entirely collapsed (**Figure 2F**). Toyoda et al. (2013a) indicated that at the inoculation site on Caliph leaves, *M. pinodes* induced scattered flecking or small necrotic lesions, which are probably associated with a local resistance response. Additionally, pycnidia were not found on Caliph (Toyoda et al., 2013a). Moussart et al. (2007) reported that 34 *Medicago* accessions, including Caliph, exhibited a high level of resistance to *M. pinodes* infection, and the lesions were limited to the inoculation site. Thus, our present observation agrees well with previous findings that extension of infection hyphae and induced lesions are confined in Caliph (Moussart et al., 2007; Toyoda et al., 2013a). Therefore, we reconfirmed again that ecotype Caliph is resistant to *M. pinodes* strain OMP-1.

### Ultrastructure of Interaction Sites between Plant and Fungus

#### Susceptible Ecotype

**FIGURE 3:**
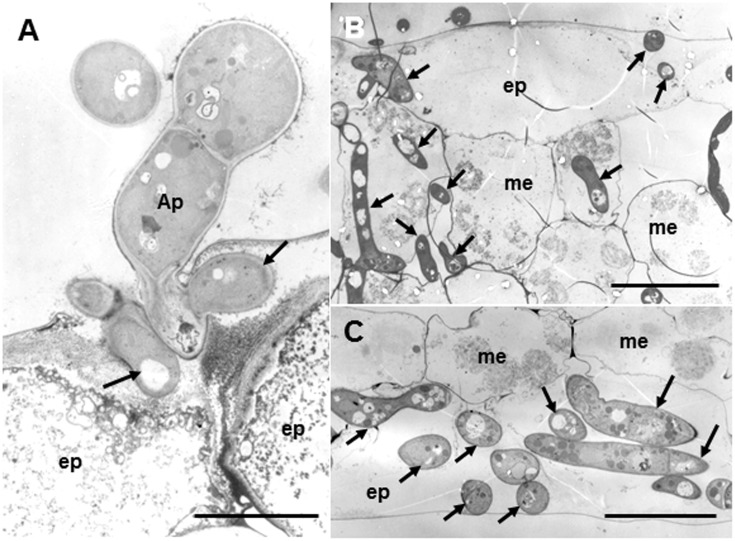
Transmission electron micrographs of susceptible R108-1 leaves at 3 days after inoculation with *M. pinodes*. **(A)** Infection hyphae (arrows) that look like infection vesicles formed in the cell wall of the adaxial epidermal (ep) cell. Appressorium (Ap). Bar = 5 μm. **(B)** Adaxial epidermis and mesophyll cells (me) invaded by hyphae. Host cell organelles were degraded. Bar = 20 μm. **(C)** Extensive hyphae in abaxial epidermal cells. Bar = 20 μm.

**Figure 3** shows fine structures of the ecotype R108-1 challenged with pycnospores of *M. pinodes* at 3 dpi, which corresponds to **Figure 2B**. Penetration pegs, which emerged from the basal part of appressoria, never directly invaded the cytoplasm of plant cells; infection hyphae (subcuticular hyphae) formed and grew in the epidermal cell walls (**Figure 3A**, arrows). Following formation of subcuticular hyphae, the hyphae proliferated in the mesophyll and abaxial epidermal cells (**Figures 3B,C**, arrows). In the invaded cells, cell organelles such as chloroplasts were entirely degraded (**Figures 3B,C**), the same as in susceptible pea epicotyls (Clulow et al., 1991). Clulow et al. (1991) observed that infection pegs of *M. pinodes* were unusually broad, and infection hyphae extended horizontally into the outer wall of epidermal cells of the epicotyls. Similarly, infection hyphae tunneled through the outer wall, creating ridges on leaf surfaces of *M. truncatula* that were visible with scanning electron microscopy (Toyoda et al., 2013a). Although many pathogenic fungi that grow subcuticularly can directly penetrate the wall of epidermal cells and grow in the cytoplasm or periplasmic space (Hohl and Stossel, 1976; Mims et al., 2000; Wharton et al., 2001), not all pathogens do. For example, as infection hyphae of *Alternaria alternata* Japanese pear pathotype or *Venturia nashicola* begin to develop, they grow into pectin layers (Park et al., 2000; Suzuki et al., 2003). Our results thus showed that infection pegs emerged from appressoria, differentiated to form an infection vesicle and the infection hyphae then subsequently grew within the outer wall of epidermis to get nutrients from the host cells. (**Figure 3A**, arrows). Similar observations were reported for *M. pinodes* by Clulow et al. (1991) and Nasir et al. (1992), respectively, indicating that infection hyphae generated from the infection vesicle tunneled through the wall of the epidermis, when the susceptible pea cultivars were challenged with the fungus. Thus, *M. pinodes* likely requires the formation of infection vesicles within the outer cell wall to enable subsequent extension of infection hyphae into the rest of the leaf tissues. During the penetration into the cuticle, the host cell wall became degraded and/or swollen near infection hyphae. This degradation and swelling of the cell wall are likely ascribed to the action of cell wall-degrading enzymes secreted by the growing hyphae. Our results also showed cell wall degradation and collapsed organelles in the leaf tissues with hyphae of R108-1. These ultrastructural changes probably result from degradative enzymes and fungal toxins known as ascochitine (Oku and Nakanishi, 1966), which are released by *M. pinodes*.

The suppressors (supprescins A and B) produced by the pycnospores of *M. pinodes* have been shown to be the major determinants of host specificity, and they certainly suppress and/or delay host defense responses (Shiraishi et al., 1992, 1997; Toyoda et al., 2016). Since the susceptibility responses of pea caused the suppressor from *M. pinodes* are well reproducible in ecotype R108-1 of *M. truncatula* (Toyoda et al., 2013a), the pycnospores also use the same strategy to establish infection in R108-1.

#### Resistant Ecotype Caliph

**FIGURE 4:**
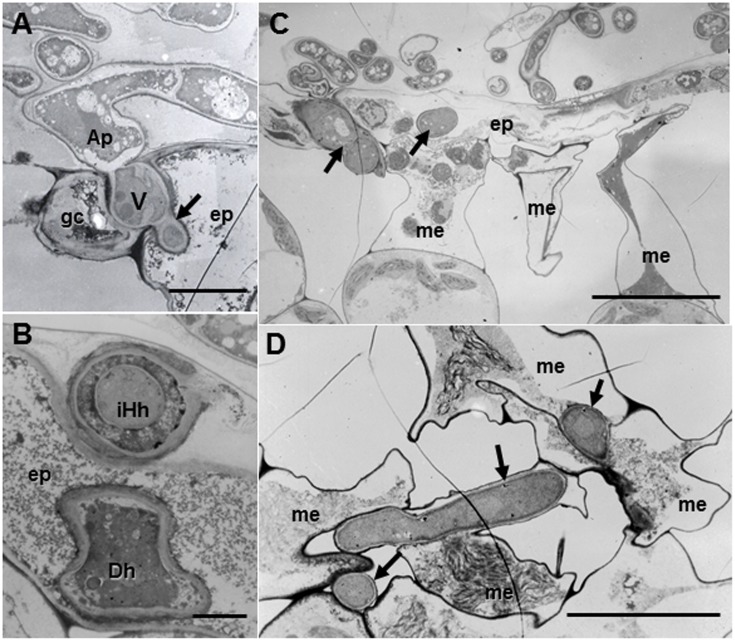
Transmission electron micrographs of resistant Caliph leaves at 3 days after inoculation with *M. pinodes*. **(A)** Infection vesicle (V) emerged from the tip of an appressorium (Ap) to cell walls between guard cell (gc) and epidermal cell (ep), and infection hyphae extended into epidermal cell (ep). Bar = 5 μm. **(B)** Aberrant hyphae in epidermal cell; intrahyphal hyphae (iHh) and dead hypha (Dh). Bar = 2 > μm. **(C)** Epidermis invaded by hyphae and adjoining mesophyll cells had shrunk. Bar = 20 μm. **(D)** Mesophyll cells in contact with the hyphae had shrunk. Bar = 10 μm.

Regardless of host ecotypes, the pycnospores of *M. pinodes* germinated and grew on the host cuticle, then penetrated the epidermal cell wall, and infection hyphae formed in the epidermal cell walls (**Figure 4A**). Although infection hyphae had extended into the palisade parenchyma by 3 days after inoculation, aberrant hyphae such as dead hyphae and intrahyphal hyphae were only found in resistant ecotype, indicating the altered fungal growth in resistant tissues (**Figure 4B**). Occasionally, hyphae were observed to pass from one epidermal cell to the next mesophyll cells, but the cytoplasmic degeneration and organelle disruption occurred in both host and fungal cells (**Figures 4C,D**).

### H_2_O_2_ Accumulation at Interaction Sites

**FIGURE 5:**
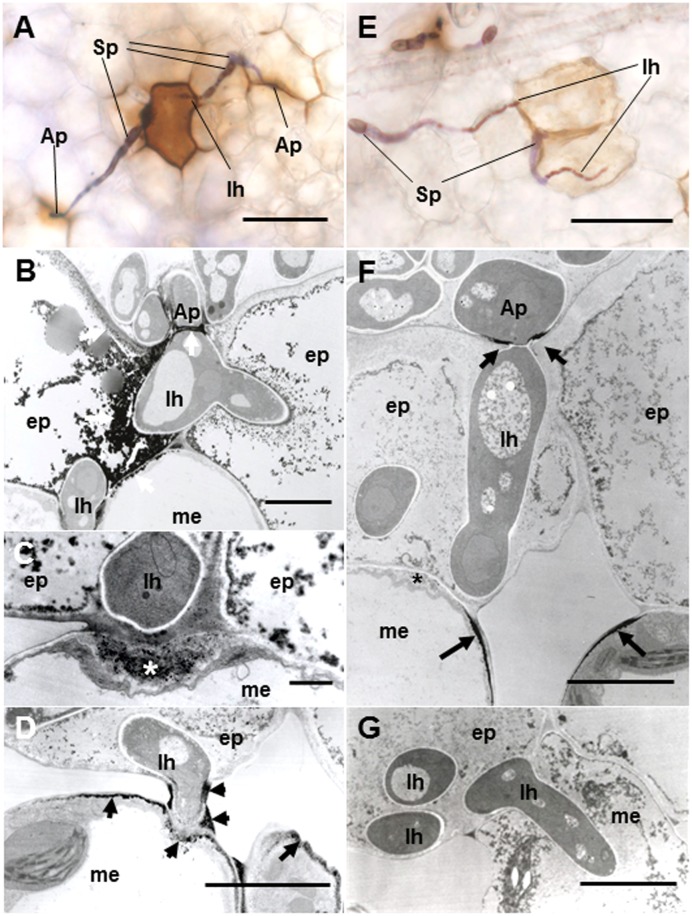
Detection of H_2_O_2_ accumulation using diaminobenzidine (DAB) **(A,E)** and cerium chloride **(B–D,F,G)** in leaves of Caliph **(A–D)** and R108-1 **(E,F)** invaded by *M. pinodes* at 18 hpi. In resistant Caliph, reddish-brown reaction product was observed where infection hyphae had formed and at the sites around appressoria **(A)**. Cerium perhydroxides deposits in cytoplasm and plasma membranes of the adaxial epidermis penetrated by hyphae, plasma membranes of mesophyll cells that abut the epidermal cells and the tip of fungal appressorium **(B)**. Cerium perhydroxides deposits inside wall apposition (asterisk, **C**), and mesophyll plasmalemma penetrated by hyphae and around hypha and its wall **(D)**. In susceptible R108-1, epidermal cells with infection hyphae were stained pale brown **(E)**. Cerium perhydroxides in the appressorial wall in contact with the epidermal wall and mesophyll plasmalemma adjacent to epidermis penetrated by hyphae **(F)**. No accumulation of cerium perhydroxides was observed where hyphae penetrated mesophyll cells **(G)**. **A,E** bars: 50 μm. **B,D,F**,**G** bars: 5 μm. **C** bars: 1 μm.

To verify resistance responses especially in Caliph, accumulation of H_2_O_2_ was analyzed *in situ* at the interaction sites using LM and TEM. Reaction of DAB with H_2_O_2_ rapidly generates insoluble, reddish-brown precipitates. In resistant ecotype Caliph, the epidermal cells where the pycnospores of *M. pinodes* attempted to penetrate or formed infection hyphae had strong reddish-brown staining (**Figure 5A**). To date, H_2_O_2_ accumulation in infected tissues has also been shown in different plant species using a cytochemical analysis with cerium chloride and TEM (Shinogi et al., 2003; Hyon et al., 2010). In the present study, the active oxygen species hydrogen peroxide (H_2_O_2_) was thus detected cytochemically through its reaction with cerium chloride to generate electron-dense deposits of cerium perhydroxides. In Caliph, the most remarkable electron-dense deposits of cerium perhydroxides were observed in epidermal cells where the pathogen invaded, and the deposits were often seen at the plasma membrane of the mesophyll cell adjacent to the epidermal cells, and at the base of the appressorium such as at the septum (**Figure 5B**, arrows). In fact, the deposits appeared in the apposition next to the host epidermal cell wall (i.e., papilla) that formed underneath the infection hyphae (**Figure 5C**, asterisk), but not in R108-1 (**Figure 5F**, asterisk). In mesophyll cells adjacent to the epidermal cells of Caliph, positive deposits appeared at the contact point between the host plasma membrane and infection hyphae and between the fungal cell wall and edge of the host cell wall (**Figure 5D**).

**FIGURE 6:**
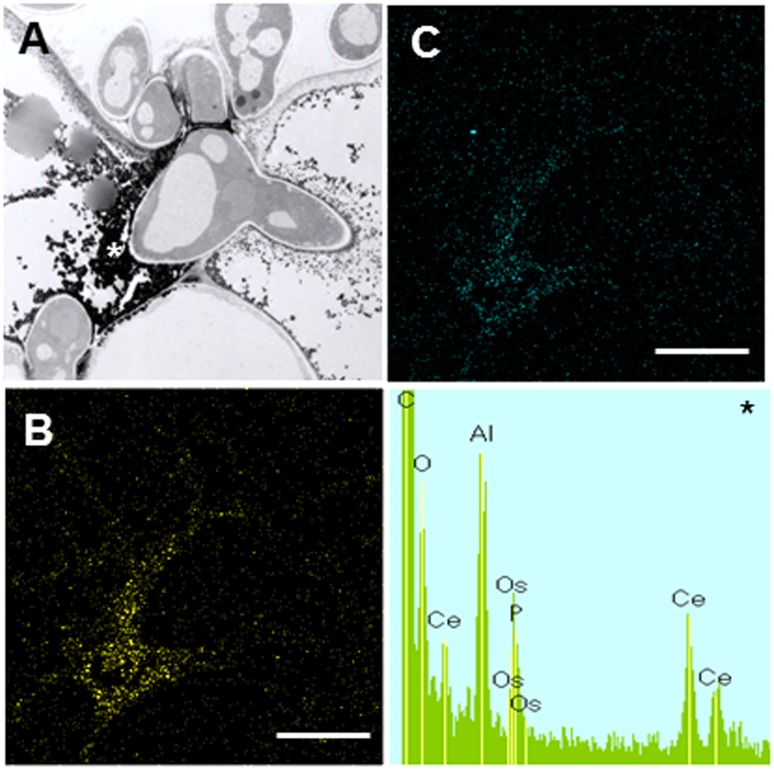
Elemental maps and energy-dispersive X-ray (EDX) spectrum showing the deposition of cerium perhydroxides at fungal invasion sites in Caliph epidermis. **(A)** Transmission electron microscopy (TEM) image is the same as **Figure 5B**. Ce **(B)** and O **(C)** mapping images, and analytical point spectrum (asterisk).

In contrast to Caliph, the epidermal cells where the pathogen penetrated or formed infection hyphae stained a very pale brown when the R108-1 was inoculated with the fungus (**Figure 5E**). At 24 hpi on R108-1, none of the epidermal cells had the strong reddish-brown staining that was present in Caliph (data not shown). Although electron-dense deposits were rarely observed in host epidermal cells, the deposits appeared only at the base of the appressorium and, sometimes at the plasmalemma of the mesophyll cell adjacent to the epidermal cell (**Figure 5F**, arrows). However, the deposits in the mesophyll plasmalemma were absent when infection hyphae were present in mesophyll cells (**Figure 5G**). These deposits were validated by STEM/EDX analysis as being from cerium perhydroxides (**Figure 6**). Highly localized production of H_2_O_2_ was found in the cytoplasm of epidermal cells penetrated by the pathogen in ecotype Caliph, but not in ecotype R108-1. In addition, H_2_O_2_ was also localized at plasma membranes and tiny deposits were found at the walls of mesophyll cells adjacent to the cell walls where infection hyphae and wall appositions were present. Taken together, our present observations indicate that a localized, intensive H_2_O_2_ accumulation is likely associated with the host defense response in the Caliph challenged with pycnospores of *M. pinodes*.

Although a small area of H_2_O_2_ was localized at the plasma membrane of mesophyll cells near the penetrating infection hyphae in R108-1 by 18 hpi, it was not present where infection hyphae had penetrated. There are many reports that fungal pathogens produce ROS and antioxidant proteins such as superoxide dismutase and catalase (Witteveen et al., 1992; Gil-ad and Mayer, 1999; Gil-ad et al., 2000; Bussink and Oliver, 2001; Mayer et al., 2001; Egan et al., 2007). Therefore, pycnospores of *M. pinodes* can likely eliminate ROS during hyphal development or suppress host defense reactions in the susceptible interaction. The ability of the fungus to generate antioxidants agrees well with our previous findings that a suppressor from *M. pinodes* effectively suppresses elicitor-stimulated O_2_^-^ and H_2_O_2_ generation, which are catalyzed by a cell wall peroxidase (Kiba et al., 1996, 1997) and copper amine oxidase (Toyoda et al., 2012), respectively. In susceptible R108-1, a tiny accumulation of H_2_O_2_ was localized underneath appressoria and around penetration pegs, probably in response to the development of these infection structures. This H_2_O_2_ generation was probably associated with fungal growth such as development of infection-related structures. In the necrotrophic Japanese pear pathotype of *A. alternata*, H_2_O_2_ accumulation was also detected at the cell walls in contact with these infection structures in both, compatible and incompatible interactions (Shinogi et al., 2003; Hyon et al., 2010). The role of ROS for fungal virulence and development has been shown in different phytopathogenic fungi (Heller and Tudzynski, 2011). Collectively, the small amount of ROS detected in R108-1 beneath the appressorium is a common phenomenon, especially in fungal pathogens that infect through the cuticle.

## Discussion

In this study, we observed infection behavior after germination of pycnospores of *M. pinodes* as well as the host cell responses to the infection, using susceptible and resistant ecotypes of *M. truncatula*. Microscopic analysis revealed that the germination and subsequent appressorial formation were commonly observed regardless of susceptible or resistant ecotype of *M. truncatula*, although the rate of formation of infection hyphae varied depending on the ecotypes (**Figure 1A**). In the susceptible ecotype R108-1, the fungus initially developed infection hyphae that grew within the outer wall of the epidermis. At 3 days after inoculation, the fungus grew intercellularly and intracellularly in subepidermal tissues of the susceptible leaves, eventually forming asexual pycnidia. Histopathological observation suggested that cell degeneration, especially mesophyll dissolution around invading hyphae is associated with expansion of disease symptoms in susceptible leaves (**Figures 2A–C**). In contrast, the rate of forming infection hyphae was considerably reduced on the resistant Caliph (**Figure 1A**), indicating an arrest in fungal growth probably due to host’s defense-related factors. Occasionally, hyphae were observed to pass from one epidermal cell to the next mesophyll cells of the Caliph (**Figures 4C,D**), but the cytoplasmic degeneration and organelle disruption occurred presumably due to a local defense elicited by the fungus. In fact, extensive production of hydrogen peroxide (H_2_O_2_) in epidermal and mesophyll cells was observed in the resistant interaction as detected by the reaction with cerium chloride to produce cerium perhydroxides under transmission electron microcopy (**Figure 4**). Our observations thus indicate that the oxidative burst leading to the generation of ROS including H_2_O_2_ is likely associated with a local defense response elicited in Caliph, since no obvious H_2_O_2_ accumulation was detectable in the susceptible R108-1.

Another striking feature of the resistant interaction was the observation of aberrant hyphae in or around the epidermal cells. Because these structures were not observed in the susceptible leaves, these aberrations may not be caused only by the structural defense. Rather, certain defense-related factors may be involved in these morphological changes of hyphae. Intrahyphal hyphae or the growth of hyphae within existing hyphae have been demonstrated in a number of phytopathogenic fungi and are considered to be survival forms under stressed conditions (Lim et al., 1983; Kim et al., 2001, 2004, 2012). Kim et al. (2001) reported that aberrant hyphal structures such as intrahyphal hyphae were found only in the resistant apple fruit tissues infected with *Botryosphaeria dothidea*. Since the intrahyphal hyphae were observed only in the Caliph, the structural modifications may not be caused solely by the structural defense. Rather, certain biochemical factors or defense-related compounds during the active defense may be involved in the hyphal morphological modifications, although the precise stimuli triggering the development of intrahyphal hyphae remain unclear. Taken together, we conclude that the structural aberrations likely are common mechanisms of fungi to be protected from a hostile environment in a resistant host by being enclosed by another hyphae. The structural differences between susceptible and resistant responses as well as the host responses will therefore provide information on defense-related characteristics of *M. pinodes* and the model host *M. truncatula*.

*Mycosphaerella pinodes* secretes a suppressor to avoid host defense responses (Shiraishi et al., 1992; Toyoda et al., 2016). In fact, the suppressor delays or suppresses elicitor-induced accumulation of *PR10-1*-mRNA in susceptible pea (Amano et al., 2013) and *M. truncatula* (Toyoda et al., 2013a). Recently, using the susceptible *M. truncatula* (ecotype R108-1), we showed the suppressor rapidly induces accumulation of mRNAs encoding almost all enzymes involved in jasmonic acid (JA) synthesis (Toyoda et al., 2013b). The application of exogenous JA to *M. truncatula* leaves evidently suppressed the elicitor-induced accumulation of *PR10-1* mRNA (Toyoda et al., 2013b). These observations indicate that a JA-mediated process(es) is probably involved in promoting susceptibility to *M. pinodes*. Plants have two major signaling molecules regulating plant immunity. Salicylic acid (SA), mediating resistance to biotrophic pathogens and JA/ethylene mediates resistance to necrotrophic pathogens (Glazebrook, 2005). In most cases, the molecules JA and SA interact with each other. Given that *M. pinodes* employs biotrophic and necrotrophic phases in its infection cycle, our results indicate that the fungus may use the JA-mediated signaling pathway through the secretion of a suppressor, to avoid the SA-regulated, elicitor-induced defenses during the early stage of infection. In our separate study with the pea, we showed that disease susceptibility to infection by *M. pinodes* was considerably reduced when *LOX* (*lipoxygenase*), *AOS* (*allene oxide synthase*), *AOC* (*allene oxide cyclase*) or *OPR* (*12-oxophytodienoic acid reductase*) were silenced (Toyoda et al., 2013b). Taken together, our results suggest that *M. pinodes* may manipulate the physiology of host cells, in particular JA synthesis, to colonize and promote disease susceptibility in the susceptible pea and *M. truncatula*.

## Conclusion

Our cytological studies on the infection process of *M. pinodes* on a susceptible and resistant ecotypes of the model plant *M. truncatula* suggested the role of the oxidative burst in host resistance. Indeed, fungal growth appears to be restricted through H_2_O_2_ production and/or associated defense-related factors. This model pathosystem involving *M. pinodes* and *M. truncatula* may assist in better understanding pathogenesis of the fungus on pea, thus providing information on the breeding of the resistant cultivars of pea.

## Author Contributions

TSu, AM, MH, TSh, and KT planned and performed the experiments. The corresponding author KT discussed the research with all authors, and TSu and KT wrote the manuscript. The final manuscript was approved by all authors.

## Conflict of Interest Statement

The authors declare that the research was conducted in the absence of any commercial or financial relationships that could be construed as a potential conflict of interest. The reviewer SM and handling Editor declared their shared affiliation, and the handling Editor states that the process met the standards of a fair and objective review.
